# Supplementary material for: Herb Robert’s Gift against Human Diseases: Anticancer and Antimicrobial Activity of Geranium robertianum L
Source: Pharmaceutics. 2023 May 22;15(5):1561. doi: 10.3390/pharmaceutics15051561 (PMC10223082; doi:10.3390/pharmaceutics15051561)
Supplement: Supplementary file 1 [file pharmaceutics-15-01561-s001.zip › pharmaceutics-2346949-supplementary.pdf]

Supplementary Materials

Article

Herb Robert's gift against human diseases – anticancer and antimicrobial activity of *Geranium robertianum* L.

Łukasz Świątek [1,*], Inga Wasilewska [2], Anastazja Boguszewska [1], Agnieszka Grzegorczyk [3], Jakub Rezmer [2], Barbara Rajtar [1], Małgorzata Polz-Dacewicz [1] and Elwira Sieniawska [4,*]

[1] Department of Virology with Viral Diagnostics Laboratory, Medical University of Lublin, Chodźki 1, 20-093 Lublin, Poland; anastazja.boguszewska@umlub.pl (A.B.); barbara.rajtar@umlub.pl (B.R.); malgorzata.polz-dacewicz@umlub.pl (M.P.-D.)
[2] Student Research Group, Department of Virology with Viral Diagnostics Laboratory, Medical University of Lublin, Chodźki 1, 20-093 Lublin, Poland; wasilewskainga@gmail.com (I.W.); jakubrezmer1@gmail.com (J.R.)
[3] Chair and Department of Pharmaceutical Microbiology, Medical University of Lublin, Chodźki 1, 20-093 Lublin, Poland; agnieszka.grzegorczyk@umlub.pl
[4] Department of Natural Products Chemistry, Medical University of Lublin, Chodźki 1, 20-093 Lublin, Poland
* Correspondence: lukasz.swiatek@umlub.pl (Ł.Ś.); esieniawska@pharmacognosy.org (E.S.)

Content

I. Tables
II. Figures
III. Materials and methods

I. Tables

Table S1. The yield of hexane fractions H1-H8

Fraction	Amount of residue [g]
H1	1.323
H2	0.507
H3	0.531
H4	0.47
H5	0.291
H6	0.131
H7	0.127
H8	0.132

Table S2. The yield of ethyl acetate fractions E1-E6

Fraction	Amount of residue [g]
E1	1.29
E2	1.23
E3	0.58
E4	0.45
E5	0.51
E6	0.58

Table S3. The phytochemical characteristics of *G. robertianum* extracts

Comp. No	R time [min]	Tentative identification	Molecular formula	Monoisotopic Mass	Pseudomolecular ion $[M-H]^-$ (m/z)	Fragment ions (m/z)	Extracts
1	2.04	Quinic acid derivative	-	534.1708	533.1708	191.0548, 173.0463	M
2	2.09	Quinic acid	C7H12O6	192.0551	191.0551	173.0031, 111.0083, 93.0347, 85.0288	M, Aq, H, EA
3	2.33	Malic acid	C4H6O5	134.0135	133.0135	114.9999, 71.0140	M, Aq, H, EA
4	2.39	Citric acid	C6H8O7	192.0236	191.0236	111.0081, 87.0089, 71.0127	M, Aq, H, EA
5	2.95	Galloylglucose isomer I	C13H16O10	332.066	331.066	169.0123, 125.0230	M, Aq
6	2.98	3-*O*-Galloylquinic acid	C14H16O10	344.0653	343.0653	191.0546, 196.0120, 125.0241, 111.0418	M
7	4.11	Gallic acid	C7H6O5	170.0139	169.0139	125.0239, 107.0115, 79.0191	M, Aq
8	4.21	4-*O*-Galloylquinic acid	C14H16O10	344.0679	343.0679	191.0548, 173.0431, 169.0131, 151.0405, 125.0229, 85.0283	M, Aq, H
9	6.24	Dihydroxybenzoic acid glucoside	C13H16O9	316.0700	315.0700	153.0199, 108.0209	M, Aq
10	7.33	Dihydroxybenzoic acid	C7H6O4	154.0182	153.0182	109.0291, 108.0298, 91.0187	M, Aq, EA
11	8.86	di-HHDP-glucose (Pedunculagin)	C34H24O22	784.0687	783.0687	481.0557, 300.9943, 275.0158, 249.0358	M, Aq
12	10.17	Caffeoylquinic acid isomer I	C16H18O9	354.0868	353.0868	191.0556, 179.0344, 161.0228, 135.0444	M, Aq, H, EA
13	11.16	Gallic acid *O*-(6-galloylglucoside)	C20H20O14	484.0772	483.0772	423.0475, 331.0623; 313.0537; 271.04118; 169.0121; 151.0003, 125.0238	M

14	11.31	Methylgallic acid	C8H8O5	184.0295	183.0295	168.0060, 124.0166, 78.0099	M
15	11.34	Dihydroxybenzoic acid pentoside	C12H14O8	286.0608	285.0608	225.0401, 153.0190, 108.0220	M, Aq
16	11.40	Galloylglucose isomer II	C13H16O10	332.0670	331.0670	169.0140, 125.0244	M, Aq
17	11.60	p-Coumaroylquinic acid	C16H18O8	338.0804	337.0804	191.0534, 163.0376	M, Aq
18	13.60	4-*O*-feruloylquinic acid	C17H20O9	368.0917	367.0917	193.0468, 173.0419	M, Aq
19	13.83	Caffeoylquinic acid isomer II	C16H18O9	354.0868	353.0868	191.0550, 179.0339, 173.0446, 135.0444	M, Aq, H, EA
20	15.03	Gallic acid pentoside	C12H14O9	302.0476	301.0476	283.0384, 169.0090, 168.0018, 125.0246	M, Aq
21	15.57	Gallacetophenone	C8H8O4	168.0361	167.0361	123.0452, 108.0224	EA
22	15.59	Castalin/Vescalin	C27H20O18	632.0568	631.0568	613.0454, 569.0471, 461.0362, 445.0386, 299.9862, 298.9837, 169.0148	Aq
23	16.30	Brevifolin	C12H8O6	248.0201	247.0201	219.0257, 191.0313, 173.0214, 145.0267	M, Aq, H, EA
24	16.72	Brevifolincarboxylic acid	C13H8O8	292.0141	291.0141	247.0230, 191.0341, 173.0246, 163.0399, 145.0295	M, Aq, H, EA
25	17.12	HDDP-galloyl-glucose (Corilagin structure)	C27H22O18	634.0723	633.0723	300.9897, 275.0112, 245.0129, 169.0125	M, Aq
26	17.97	Ellagitannin	-	816.0582	815.0582	797.0130, 753.0277, 725.0313, 475.0231 300.9909, 249.0346	M
27	19.21	Geraniin	C41H28O27	952.0563	951.0563	933.0448, 915.0304, 463.0440, 300.9923, 169.0091	M, Aq, H

28	19.71	Methyl brevifolincarboxylate	C14H10O8	306.0304	305.0304	273.0033, 245.0084, 217.0137, 173.0210, 161.0239, 145.0279, 133.0269, 117.0326, 105.0329	M
29	20.97	4-*O*-galloylchlorogenic acid	C23H22O13	506.0507	505.0507	353.0538, 191.0374, 179.0173, 168.9952, 135.0315	M, Aq, H
30	22.38	Flavonoid derivative		740.2087	739.2087	575.1357, 473.1003, 327.0465, 274.0292, 255.0242, 178.9957. 151.0033	Aq
31	22.39	Tetragalloylglucose isomer	C34H28O22	788.0811	787.0811	635.0021, 617.0193, 465.0292, 447.0157, 300.9686, 246.9985, 168.9956	M
32	22.40	Caffeic acid derivative		296.0080	295.0080	251.0155, 207.0260, 179.0309, 163.0370, 135.0433	M, Aq
33	22.47	Ellagic acid pentoside	C19H14O12	434.0420	433.0420	300.9688; 299.9590	M
34	23.16	Rutin	C27H30O16	610.1321	609.1321	463.0792, 301.0277, 300.0212, 271.0186, 178.9955	M, Aq, H
35	23.36	Kaempferol-3-*O*- rutinoside	C27H30O15	594.1525	593.1525	285.0137, 257.0148, 255.0101	M, Aq, H
36	23.66	Ellagic acid	C14H6O8	302.0025	301.0025	283.9679, 244.9839, 228.9909, 200.9985, 173.0067, 157.0131, 145.0145	M, Aq, H, EA
37	23.94	Ellagic acid hexoside	C20H16O13	464.0475	463.0475	300.9715	M, Aq, H
38	24.25	HDDP-galloyl-glucose based structure	C41H28O28	970.0838	969.0838	925.0539, 881.0645, 755.0491, 711.0612, 633.0522, 463.0409, 300.9915, 247.0202, 169.0120	Aq
39	24.73	Pentagalloylglucose isomer I	C41H32O26	940.1109	939.1109	769.0298, 617.0333, 465.0149, 447.0233, 341.0305, 169.0014	M
40	27.46	Kaempferol-3-*O*- glucoside	C21H20O11	448.0943	447.0943	285.0189, 284.0096, 255.0020, 150.9886	M, Aq

41	30.06	Fatty compound	C12H24O2	200.1324	199.1324	125,0941	H
42	30.92	Quercetin	C15H10O7	302.0291	301.0291	257.0401, 178.9959, 151.0023	M, Aq, H, EA
43	32.09	Fatty compound	C18H32O5	328.212	327.212	-	M, Aq, H, EA
44	32.34	Undecanedioic acid	C11H20O4	216.1328	215.1328	153.1287, 197.1177	EA
45	33.68	Fatty compound	C18H34O5	330.234	329.0234	-	M, Aq, H, EA
46	34.35	Kaempferol	C15H10O6	286.0401	285.0401	151.0033	M, Aq, H, EA
47	34.56	Unidentified	C7H6O5	170.0141	169.0141	125.0242, 151.0033	EA
48	34.61	Kaempferol derivative	-	344.0884	343.0884	209.1152, 285.0445	H
49	35.15	Fatty compound	C16H32O4	288.2272	287.2272	-	EA
50	41.35	Fatty compound	C18H30O4	310.2058	309.2058	171.1039, 277.1813, 291.1961	H
51	46.00	Unidentified	C20H28O3	316.1989	315.1989	297.1862; 271.2084	H
52	46.57	Unidentified	C18H28O2	276.2017	275.2017	231.2102	H
53	46.95	Hexadecanedioic acid	C16H30O4	286.2092	285.2092	108.7677, 223.2066, 267.1964	H
54	47.24	Fatty compound	C18H30O3	294.2148	293.2148	275.2010; 224.1404; 195.1381	H
55	48.33	Unidentified	C16H26O12	410.1337	409.1337	264.0462, 283.0310, 363.0917	H
56	49.41	Fatty compound	C18H32O3	296.2284	295.2284	277.2165, 195.1378	M, Aq, H, EA
57	50.92	Fatty compound	C18H32O4	312.1990	311.1990	153.1259, 185.1165, 249.2207, 293.3111	H

M- methanolic, Aq- aqueous, H- hexane, EA- ethyl acetate. HHDP – hexahydroxydiphenoyl, DHHDP – dehydrohexahydroxydiphenoyl. The identification was based on the PubChem database and the following literature sources: [1-5]

II. Figures

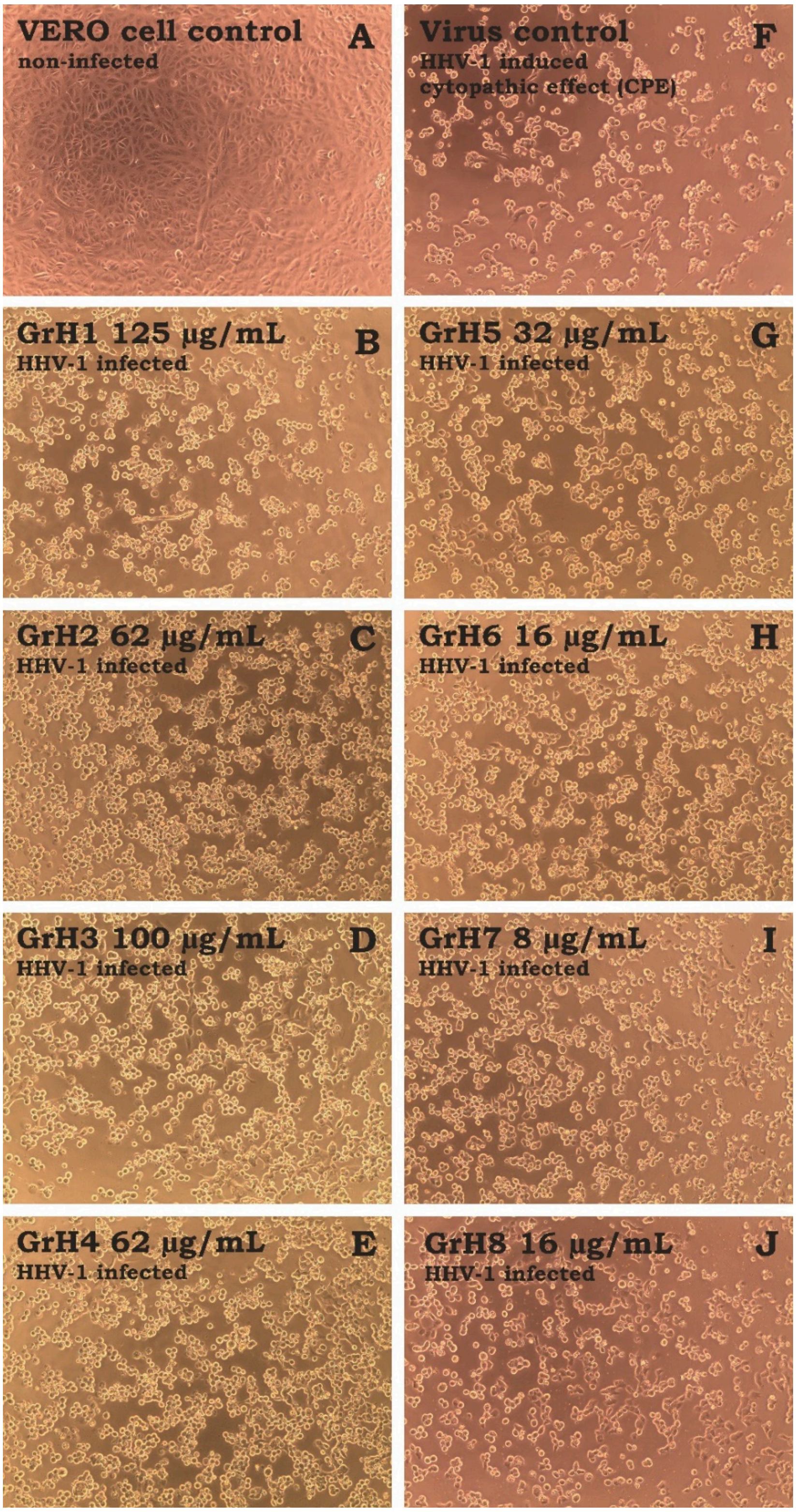

Figure S1. Influence of fractions obtained from *G. robertianum* hexane extract on the development of HHV-1-induced CPE. (A – VERO cell control; HHV-1 infected VERO cells treated with GrH1 125 µg/mL (B), GrH2 62 µg/mL (C), GrH3 100 µg/mL (D), GrH4 62 µg/mL (E), GrH5 32 µg/mL (G), GrH6 16 µg/mL (H), GrH7 8 µg/mL (I), GrH8 16 µg/mL (J); F – Virus control, HHV-1 infected and non-treated VERO cells)

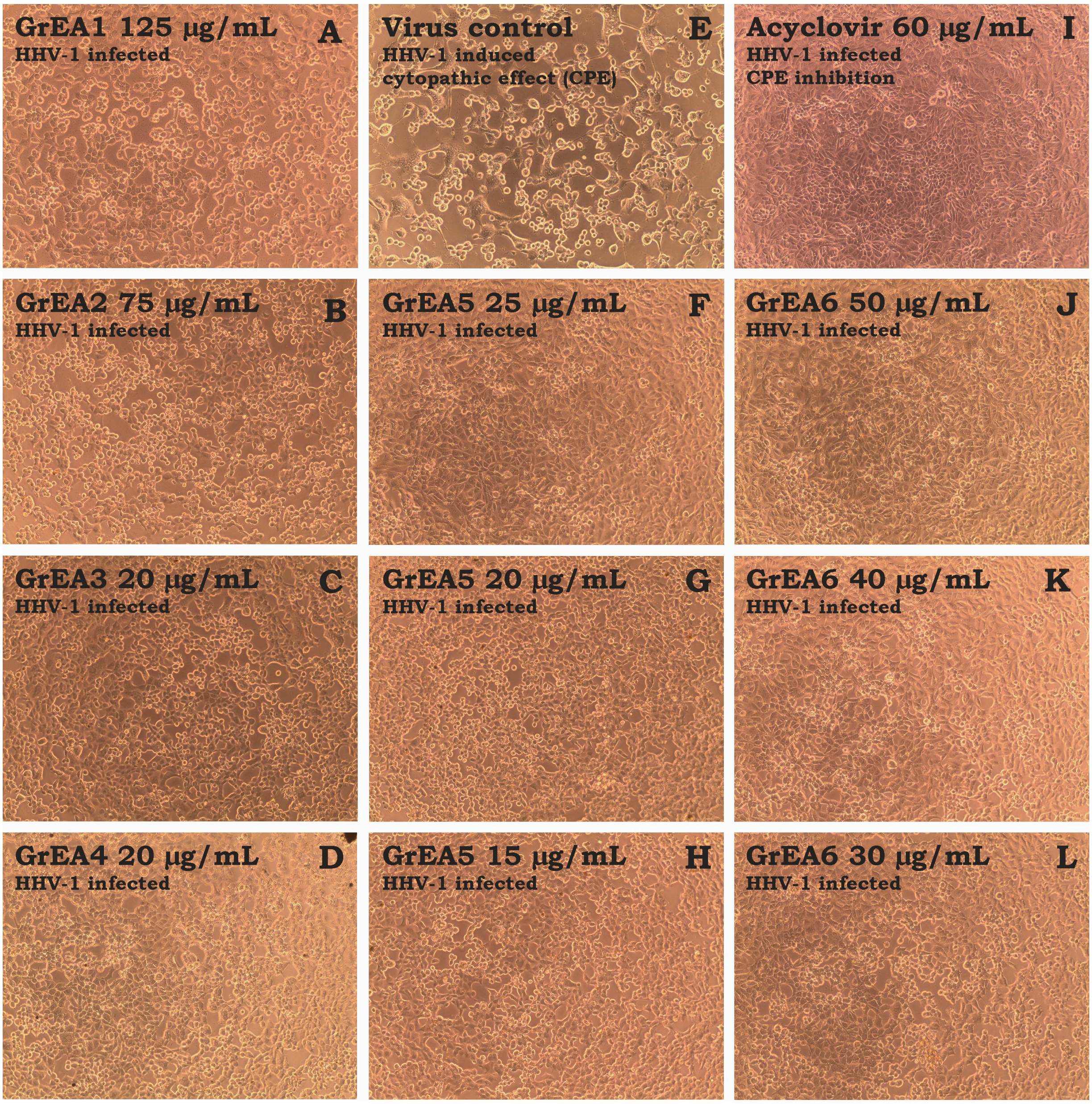

Figure S2. Inhibition of the CPE development by fractions obtained from *G. robertianum* ethyl acetate extract and acyclovir. (HHV-1 infected VERO cells treated with GrEA1 125 µg/mL (A), GrEA2 75 µg/mL (B), GrEA3 20 µg/mL (C), GrEA4 20 µg/mL (D), GrEA5 25 µg/mL (F), GrEA5 20 µg/mL (G), GrEA5 15 µg/mL (H), Acyclovir 60 µg/mL (I), GrEA6 50 µg/mL (J), GrEA6 40 µg/mL (K) GrEA6 30 µg/mL L); E – Virus control, HHV-1 infected and non-treated VERO cells)

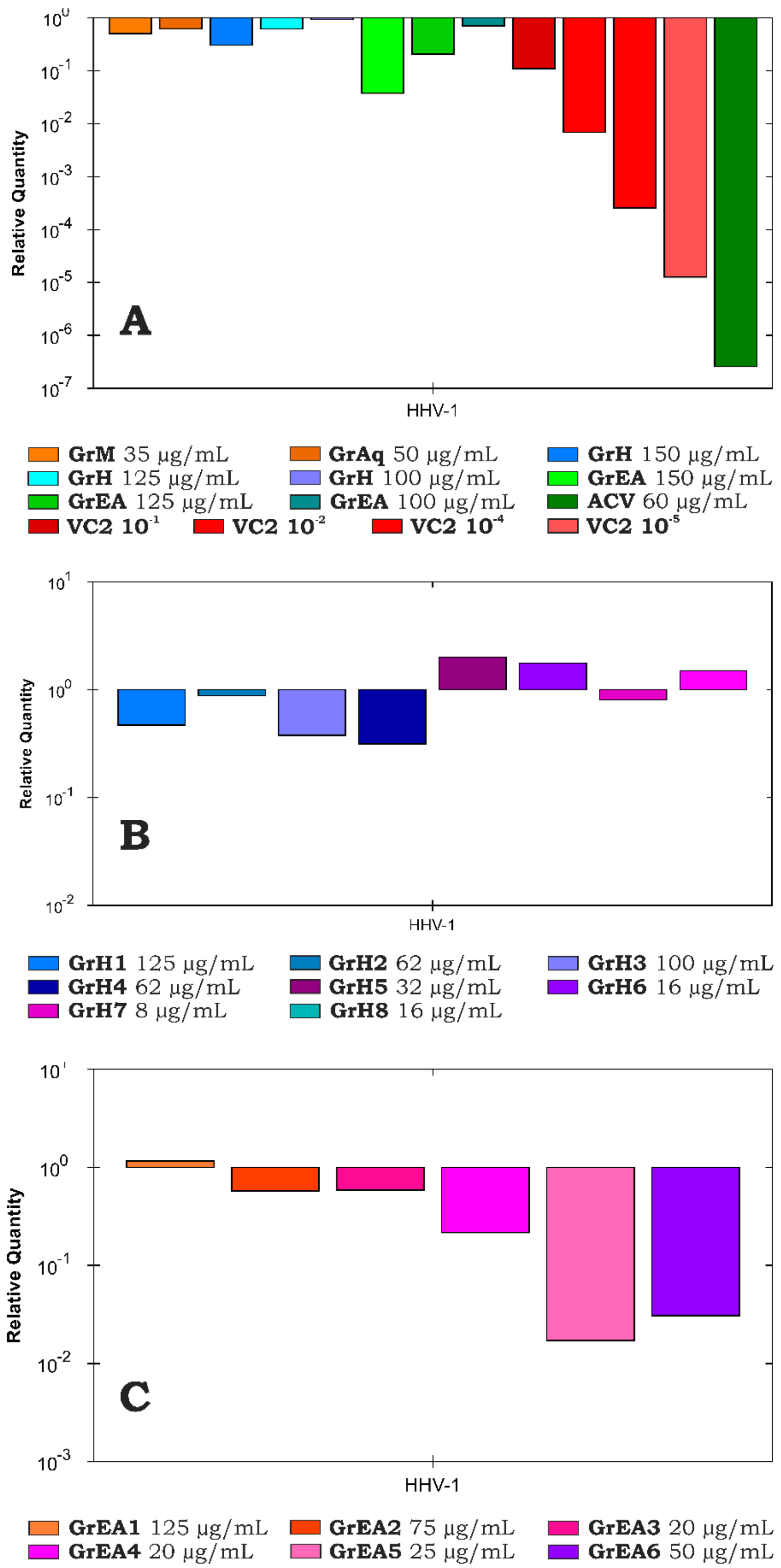

Figure S3. The assessment of HHV-1 viral load in the tested samples in relation to virus control based on the relative quantity (ΔCq) method. (Reduction of HHV-1 viral load by: A – crude extracts, B – fractions from hexane extract, C – fractions from ethyl acetate extract)

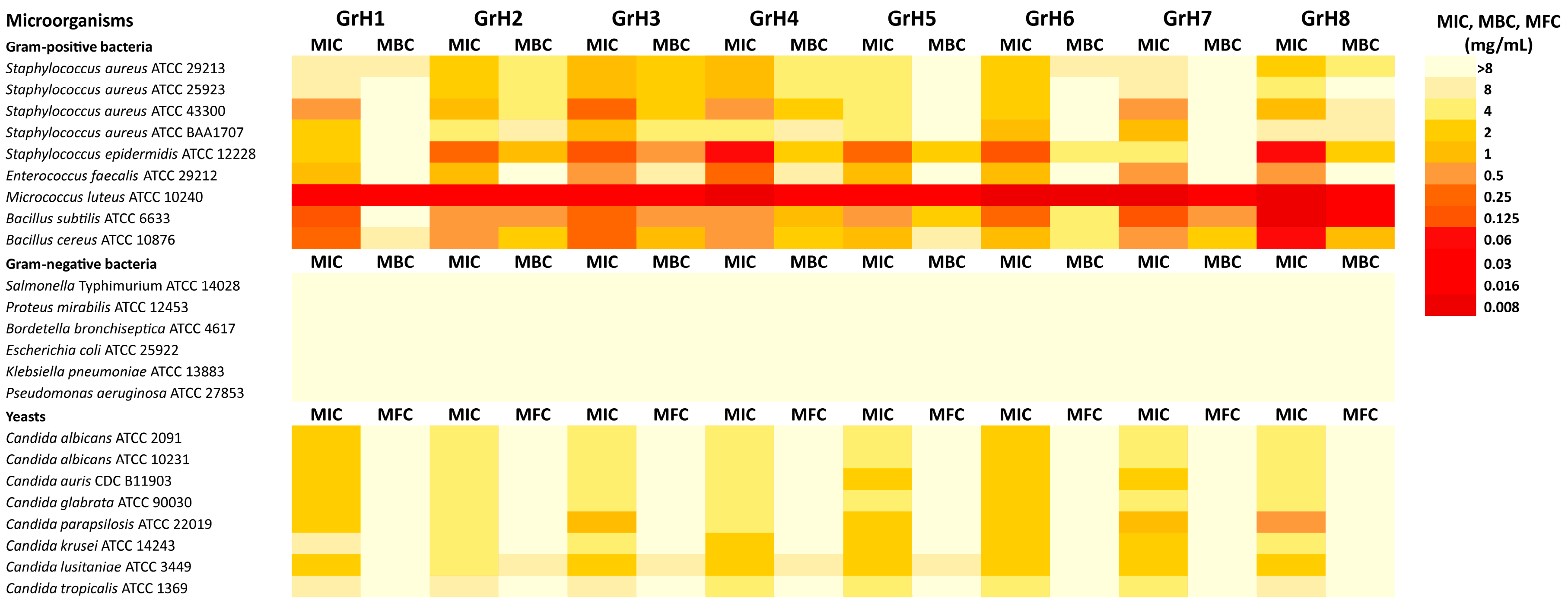

Figure S4. The antimicrobial potential of fractionations obtained from *G. robertianum* hexane extract (MIC – Minimum Inhibitory Concentration (mg/mL); MBC − Minimum Bactericidal Concentration (mg/mL); MFC – Minimum Fungicidal Concentration (mg/mL); reference antimicrobial substances MIC values: fluconazole 1 µg/mL for *Candida albicans* ATCC 10231, vancomycin 1 µg/mL for *Staphylococcus aureus* ATCC 29213, and ciprofloxacin 0.015 µg/mL for *Escherichia coli* ATCC 25922)

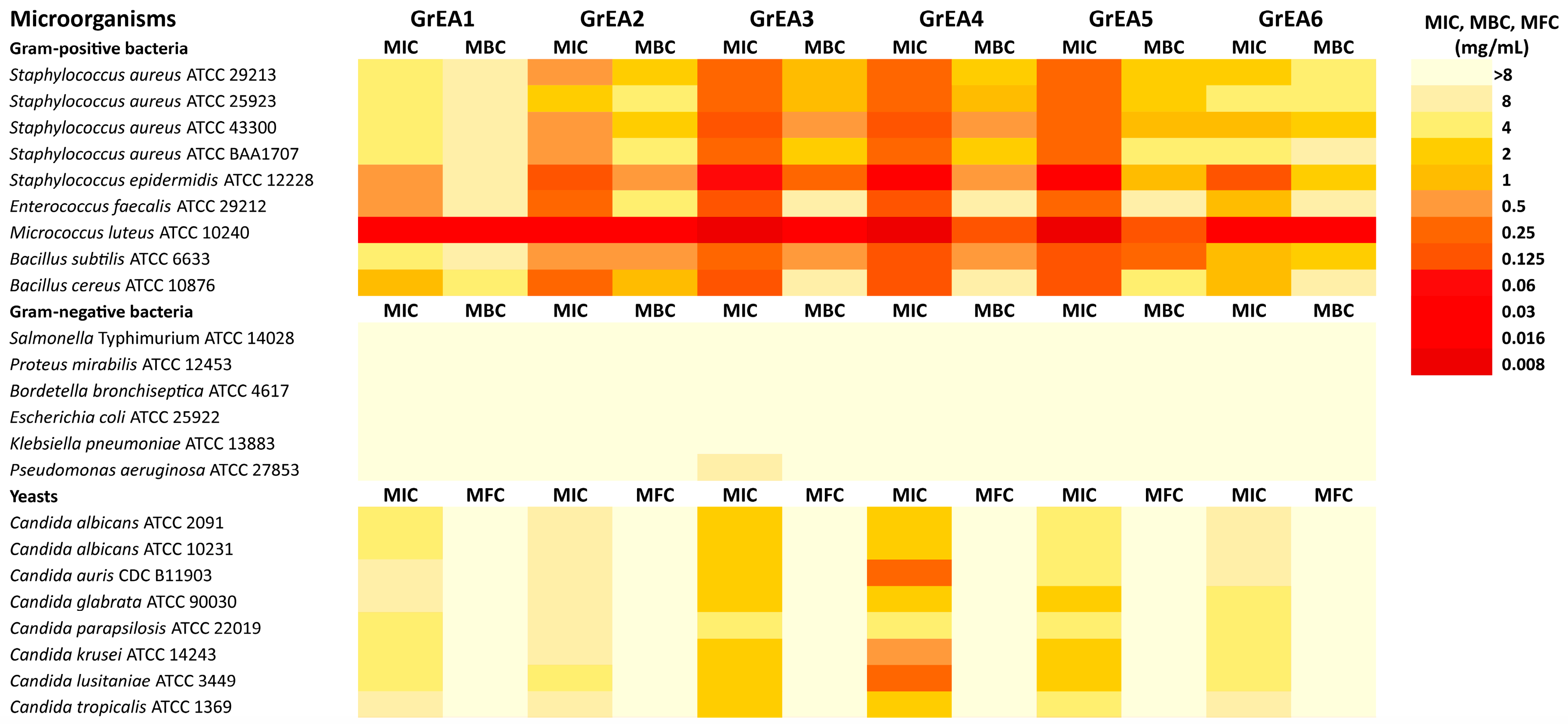

Figure S5. The antimicrobial activity of fractionations obtained from *G. robertianum* ethyl acetate extract (MIC – Minimum Inhibitory Concentration (mg/mL); MBC – Minimum Bactericidal Concentration (mg/mL); MFC – Minimum Fungicidal Concentration (mg/mL); reference antimicrobial substances MIC values: fluconazole 1 µg/mL for *Candida albicans* ATCC 10231, vancomycin 1 µg/mL for *Staphylococcus aureus* ATCC 29213, and ciprofloxacin 0.015 µg/mL for *Escherichia coli* ATCC 25922)

III. Materials and methods

Cell line maintenance and in vitro experiments

The cytotoxicity of *G. robertianum* crude extracts (aqueous, methanolic, ethyl acetate and hexane), as well as fractions obtained from ethyl acetate extract (GrEA1-GrEA6) and hexane extract (GrH1-GrH8), was evaluated *in vitro* towards normal VERO (ATCC, CCL-81) cells and cancer-derived cell lines – FaDu (ATCC, HTB-43, human hypopharyngeal squamous cell carcinoma), Detroit 562 (human pharyngeal carcinoma, ATCC, CCL-138), and RKO (human colon cancer, ATCC, CRL-2577) using 3-(4,5-dimethylthiazol-2-yl)-2,5-diphenyltetrazolium bromide (MTT) based protocol.

Media used for *in vitro* culturing included Dulbecco Modified Eagle Medium (DMEM, Corning, Tewksbury, MA, USA) used for VERO cells and Modified Eagle Medium (MEM, Corning) used for cancer-derived cell lines. Cell media used in the experiments were supplemented with antibiotics (Penicillin-Streptomycin Solution, Corning) and fetal bovine serum (FBS, Corning) – 10% (cell passaging) and 2% (cell maintenance and experiments). Phosphate buffered saline (PBS) and trypsin were bought from Corning, whereas MTT (3-(4,5-dimethylthiazol-2-yl)-2,5-diphenyltetrazolium bromide) and DMSO (dimethyl sulfoxide) from Sigma (Sigma-Aldrich, St. Louis, MO, USA). Incubation was carried out in a 5% CO_2 atmosphere at 37°C (CO_2 incubator, Panasonic Healthcare Co., Tokyo, Japan).

Concentrations of cells for passaging were as follows: VERO – $1.5*10^5$ cells/mL, FaDu – $2.5*10^5$ cells/mL, Detroit 562 – $2*10^5$ cells/mL, and RKO – $4*10^5$ cells/mL.

Stock solutions of extracts were prepared by dissolving the extracts in cell culture grade DMSO (PanReac Applichem). Stock solutions of extracts were stored frozen (-23°C) until used.

Evaluation of cytotoxicity

Cytotoxicity was tested using an MTT-based protocol following a previously described protocol [3]. Briefly, the cells were passaged into 96-well plates (Falcon, TC-treated, Corning) and, after overnight incubation, treated with serial dilutions of extract or fraction stock solutions for 72 h. Simultaneously, the cytotoxicity of DMSO in concentrations equal to those present in the dilutions of stock solutions was tested to exclude any effect on the assay. Afterwards, the media was removed, cells were washed with PBS, and 10% of MTT solution

(5 mg/mL) in cell media was added, and the incubation continued for the next 4 h. Subsequently, the SDS/DMF/PBS (14% SDS, 36% DMF, 50% PBS) solvent was used (100 μL per well) to dissolve the precipitated formazan crystals, and the plates were left at 37°C overnight. Finally, the Synergy H1 Multi-Mode Microplate Reader (BioTek Instruments, Inc. Winooski, Vermont, USA) with Gen5 software (ver. 3.09.07; BioTek Instruments, Inc.) was used to measure the absorbance (540 and 620 nm).

Evaluation of the antiviral properties

The antiviral activity of *G. robertianum* extracts and fractions was tested against HHV-1 (ATCC, Cat. No. VR-260) propagated in the VERO cell line. The antiviral assays involved the influence of extracts on the formation of virus-induced cytopathic effect (CPE) and the semi-quantitative assessment of the viral load using Real-Time PCR.

Evaluation of the influence on the virus-induced CPE

The infectious titer of HHV-1 used in this study was 5.5±0.25 log$CCID_{50}$/mL ($CCID_{50}$ – 50% cell culture infectious dose). Briefly, the VERO cells (monolayer) in 48-well plates (Falcon, clear flat bottom TC-treated, Corning) were treated (500 μL/well) with HHV-1 suspension (100-fold $CCID_{50}$/mL) in cell media and incubated for 1 hour, leaving at least two uninfected wells as VERO cell control. Afterwards, the media were removed, monolayers washed with PBS, and the non-toxic concentrations of extracts or fractions, the highest concentration not exceeding the CC_{10} values, diluted in cell media were added. The non-infected VERO cells (cell control) and non-treated infected cells (virus control) wells were maintained in media containing 2% FBS. The incubation was conducted until cytopathic effect (CPE) was observed (inverted microscope CKX41, Olympus Corporation, Tokyo, Japan) in virus control, usually approx. 72h. Afterwards, the plates were observed for possible inhibition of CPE by tested extracts compared to the CPE in virus control, and the results were recorded. Lastly, the plates were thrice frozen (-72°C) and thawed; the samples were collected and stored at -72°C until DNA isolation [3].

Real-Time PCR for HHV-1 viral load

The DNA isolation was carried out using a commercially available kit (QIAamp DNA Mini Kit, Cat#51304, QIAGEN GmbH, Hilden, Germany) following the manufacturer's

instructions. The Real-Time PCR amplification was performed using SybrAdvantage qPCR Premix (Takara Bio Inc., Kusatsu, Shiga Prefecture, Japan) and primers (UL54F – 5′ CGCCAAGAAAATTTCATCGAG 3′, UL54R – 5′ ACATCTTGCACCACGCCAG 3′) on the CFX96 thermal cycler (Bio-Rad Laboratories, Inc., California, USA). The amplification cycle parameters were as follows: initial activation (95°C, 20 secs); cycling (40 repeats: denaturation (95°C, 5 secs), annealing and synthesis (60°C, 30 secs), fluorescence acquisition); melting curve analysis (65-95°C). The HHV-1 viral load in the tested samples was assessed in relation to virus control based on the relative quantity (ΔCq) method using CFX Manager™ Dx Software (Bio-Rad Laboratories).

Supplementary material references

1. Catarino, M. D.; Silva, A. M. S.; Cruz, M. T.; Cardoso, S. M., Antioxidant and anti-inflammatory activities of *Geranium robertianum* L. decoctions. *Food & Function* **2017,** 8, (9), 3355-3365.
2. Graça, V. C.; Barros, L.; Calhelha, R. C.; Dias, M. I.; Ferreira, I. C. F. R.; Santos, P. F., Bio-guided fractionation of extracts of *Geranium robertianum* L.: Relationship between phenolic profile and biological activity. *Industrial Crops and Products* **2017,** 108, 543-552.
3. Świątek, Ł.; Sieniawska, E.; Sinan, K. I.; Maciejewska-Turska, M.; Boguszewska, A.; Polz-Dacewicz, M.; Senkardes, I.; Guler, G. O.; Bibi Sadeer, N.; Mahomoodally, M. F.; Zengin, G., LC-ESI-QTOF-MS/MS Analysis, Cytotoxic, Antiviral, Antioxidant, and Enzyme Inhibitory Properties of Four Extracts of *Geranium pyrenaicum* Burm. f.: A Good Gift from the Natural Treasure. *Int J Mol Sci* **2021,** 22, (14).
4. Sieniawska, E.; Świątek, Ł.; Sinan, K. I.; Zengin, G.; Boguszewska, A.; Polz-Dacewicz, M.; Bibi Sadeer, N.; Etienne, O. K.; Mahomoodally, M. F., Phytochemical Insights into *Ficus sur* Extracts and Their Biological Activity. *Molecules* **2022,** 27, (6).
5. Singh, A.; Bajpai, V.; Kumar, S.; Sharma, K. R.; Kumar, B., Profiling of Gallic and Ellagic Acid Derivatives in Different Plant Parts of *Terminalia arjuna* by HPLC-ESI-QTOF-MS/MS. *Natural Product Communications* **2016,** 11, (2).
